# Associations between physical activity and adolescent well-being: a loneliness-moderated chain mediation model with explainable machine learning

**DOI:** 10.3389/fpsyg.2026.1873426

**Published:** 2026-08-27

**Authors:** Longjiang Chen, Haoyi Tan, Zixiong Sheng, Tian Yin, Jingyu Chen, Lan Luo

**Affiliations:** 1 College of Physical Education, Yunnan Normal University, Kunming, China; 2 School of Public Affairs, Zhejiang University, Hangzhou, China; 3 Business School, NingboTech University, Ningbo, China; 4 School of Physical Education, Yunnan Minzu University, Kunming, China; 5 School of Sports Economics and Management, Xi'an Physical Education University, Xi’an, China; 6 School of Physical Education and Health, Sichuan Technology and Business University, Meishan, China

**Keywords:** loneliness, machine learning, physical activity, psychological resilience, well-being

## Abstract

**Introduction:**

This cross-sectional survey examined how physical activity was associated with adolescent well-being through cognitive reappraisal and psychological resilience, and whether these associations varied by loneliness.

**Methods:**

Data from 964 adolescents aged 10–19 years were analyzed using moderated chain mediation models. XGBoost regression and SHAP interpretation were used as supplementary predictive tools.

**Results:**

Physical activity showed no significant direct association with well-being after cognitive reappraisal and psychological resilience were included. Significant indirect associations were observed through cognitive reappraisal, through psychological resilience, and through the sequential pathway linking cognitive reappraisal with psychological resilience. Loneliness moderated the association between psychological resilience and well-being, with a stronger positive association among adolescents reporting higher loneliness. The XGBoost model showed acceptable internal predictive performance in the test set (RMSE = 2.599, MAE = 1.860, R² = 0.640). SHAP analysis indicated that psychological resilience made the largest predictive contribution, followed by loneliness and cognitive reappraisal, whereas physical activity made a smaller contribution. Partial dependence results suggested a modest nonlinear pattern for physical activity, with predicted well-being increasing mainly from low to moderate activity levels and then tending to plateau.

**Discussion:**

Overall, the findings suggest that physical activity is linked to adolescent well-being mainly through psychological resources. The observed associations should be interpreted as cross-sectional and predictive patterns rather than causal effects.

## 1 Introduction

Adolescence is a formative developmental period in which biological, psychological, and social changes shape later health and adjustment. Mental health difficulties during this stage can affect school functioning, social relationships, and well-being across the life course (Morris, 2001). Globally, approximately one in seven adolescents aged 10–19 years experiences a mental disorder, and depression, anxiety, and behavioural disorders are among the leading causes of illness and disability in this age group (World Health Organization, 2025). Evidence from Chinese adolescent samples also indicates that well-being and psychological symptoms are closely embedded in family, school, socioeconomic, and lifestyle contexts (Zhou et al., 2018; Zhou et al., 2020). These findings make adolescent well-being an important issue for psychology, education, and public health, particularly in educational contexts where developmental stressors and health-related behaviours may jointly shape positive adjustment.

Physical activity has been widely discussed as a low-cost and accessible behavior related to adolescent mental health and well-being (Biddle et al., 2019; Wang et al., 2025). Existing evidence suggests that adolescents who engage in higher levels of physical activity often report more favorable psychological outcomes. Recent meta-analytic findings indicate that physical exercise is associated with adolescents’ physical health, mental health, and social adaptation, including lower depressive symptoms, higher self-esteem, and low-to-moderate differences in well-being (Bengtsson et al., 2025). Similarly, evidence from Danish adolescents showed that participation in organized recreational sports was related to higher levels of well-being across health-related quality-of-life dimensions (Meiner et al., 2025). These findings indicate a meaningful link between physical activity and adolescent well-being, but they also raise an important theoretical question: which psychological resources are most closely involved in this association, and under what interpersonal conditions might this association become more pronounced? Addressing this question may help refine the psychological understanding of how health-related behaviors and positive development are connected during adolescence. However, the association between physical activity and adolescent well-being is not uniformly strong across studies. Its magnitude may vary according to the type, intensity, and context of physical activity, as well as the psychological and social resources that accompany activity participation. Therefore, physical activity may be better understood not only as a behavioral correlate of well-being, but also as a context in which proximal psychological resources, such as adaptive emotion regulation and resilience, may be linked to adolescent well-being.

Cognitive reappraisal (CR), as a core strategy of emotion regulation, refers to the process by which individuals adjust their emotional responses by altering their cognitive interpretations of emotion-inducing events (Gross, 1998). According to Gross’s process model of emotion regulation, cognitive reappraisal is an antecedent-focused strategy that occurs before the full emotional response unfolds and can shape subsequent emotional experience and expression. Extensive research confirms that individuals who frequently use cognitive reappraisal experience more positive emotions and fewer negative emotions, and report higher levels of life satisfaction and psychological well-being (Cutuli, 2014; Hu et al., 2014). Psychological resilience, on the other hand, is an individual’s adaptive capacity to successfully cope with adversity, trauma, or significant stress (Masten, 2014), and is regarded as a core construct of positive psychology. Individuals with high psychological resilience are able to “bounce back” when faced with challenges and even grow from adversity (Tugade and Fredrickson, 2004). Longitudinal studies indicate that psychological resilience is a key protective factor against psychopathological risks and a promoter of positive developmental outcomes (Zimmerman et al., 2013). Theoretical analysis suggests that a sequential relationship may exist between cognitive reappraisal and psychological resilience. Specifically, cognitive reappraisal may serve as a foundational ability for the development of psychological resilience; individuals who can flexibly reframe their perceptions of negative situations are better able to maintain adaptive functioning when facing adversity, thereby exhibiting higher levels of psychological resilience. Empirical research supports this inference. Liu et al. (2024) found that cognitive reappraisal and psychological resilience sequentially mediated the association between family support and social anxiety. Feng and Wu (2024) also reported a sequential mediating role of cognitive reappraisal and psychological resilience in the association between implicit beliefs about emotion and psychological well-being. This chain-mediation framework is also theoretically relevant in the context of sport and physical activity. Physical activity settings often involve fatigue, frustration, competition pressure, and performance errors, all of which may require adolescents to reinterpret challenging experiences in more adaptive ways (Jones and Sheffield, 2007). In this sense, adolescents who report greater participation in physical activity may also report more frequent use of cognitive reappraisal. Cognitive reappraisal, in turn, is conceptually related to psychological resilience because flexible reinterpretation of stressful events may accompany more adaptive coping and recovery from difficulties. Therefore, cognitive reappraisal and psychological resilience were examined as a theoretically meaningful psychological pathway through which physical activity may be associated with adolescents’ well-being.

Loneliness (LN) refers to the distressing experience that arises when perceived social relationships fall short of desired social relationships (Perlman and Peplau, 1981). As a form of perceived social disconnection, loneliness is closely related to adolescent mental health and well-being. Epidemiological studies suggest that many adolescents experience loneliness at some point in development (Qualter et al., 2015). Social disruptions during the post-pandemic period may have further increased the salience of loneliness, as reduced face-to-face interaction, increased reliance on online learning, and fewer real-life social opportunities may intensify perceived social disconnection (Loades et al., 2020). A meta-analysis by Maes et al. (2019) showed that loneliness is associated with depressive symptoms, anxiety, and lower life satisfaction across cultural contexts. Loneliness may also shape the role of protective psychological resources. For example, Skoko et al. (2024) found that self-esteem can buffer the negative association between loneliness and mental health, suggesting that the protective function of individual resources may vary across levels of loneliness. Therefore, loneliness may moderate the influence of psychological resilience on well-being. From a theoretical perspective, the adaptive function of psychological resilience may be more pronounced when external social resources are scarce. When adolescents perceive sufficient social connections and support, the role of psychological resilience in maintaining well-being may be relatively minor, as they can rely on external resources to cope with challenges. However, when adolescents feel lonely and lack social support, the importance of internal psychological resources in protecting well-being becomes more prominent. In other words, loneliness may amplify the positive effects of psychological resilience on well-being, exhibiting a compensatory or protective-enhancing moderating pattern (Cohen and Wills, 1985).

**Figure 1:**
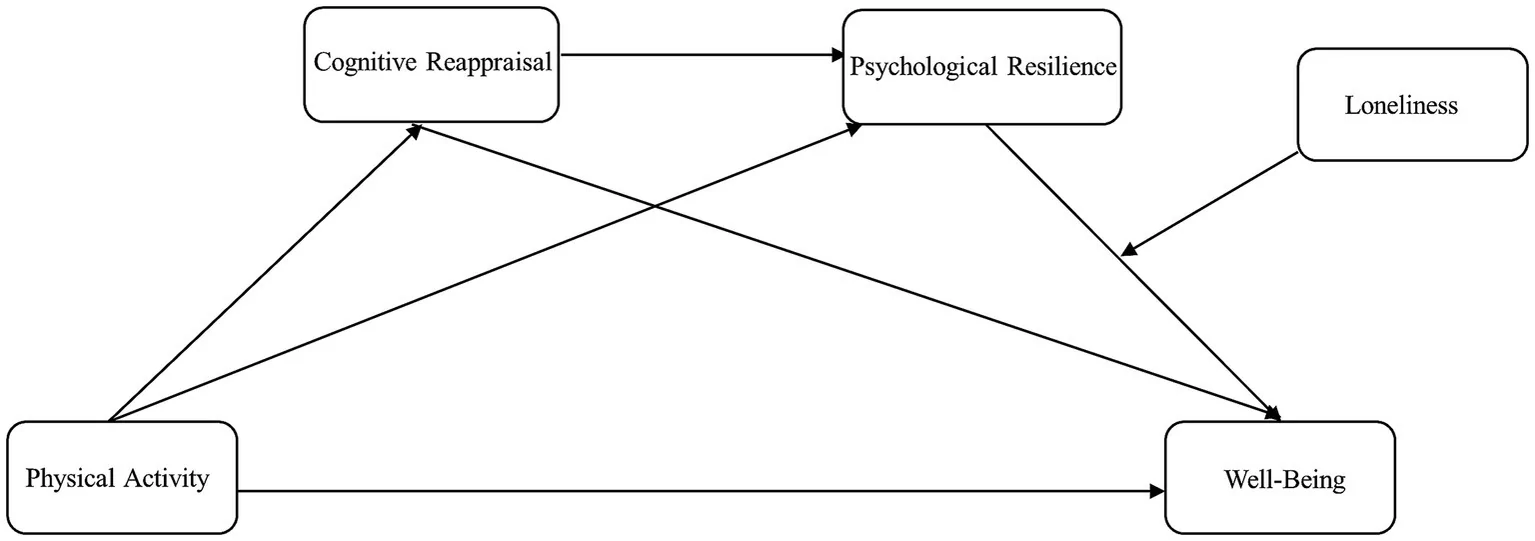
Schematic diagram of the hypothetical model.

Although the proposed moderated chain mediation model is grounded in prior theory, few studies have examined whether cognitive reappraisal and psychological resilience statistically account for the association between physical activity and well-being in sequence, or whether loneliness conditions the latter part of this pathway. Addressing this gap may clarify how lifestyle behaviors are linked to positive psychological functioning during adolescence. In addition, identifying boundary conditions may help future research determine for whom and under what psychosocial conditions physical activity is more strongly associated with well-being. Because these associations may also involve nonlinear and conditional patterns, supplementary XGBoost and SHAP analyses were used to describe the broader predictive structure of well-being and the model-based contribution of each predictor. Recent studies in physical activity and adolescent health have increasingly used machine learning to complement traditional statistical models when the aim is to classify movement behaviors, describe heterogeneous predictive patterns, or compare the relative contribution of multiple behavioral and psychosocial factors (Kadirvelu et al., 2025; Sameh et al., 2025; Thornton et al., 2022). XGBoost provides a flexible tree-boosting framework for modeling nonlinear predictive associations (Chen and Guestrin, 2016), while SHAP offers a unified approach for interpreting feature contributions to model predictions (Lundberg and Lee, 2017). In the present study, these methods were used as supplementary predictive tools rather than as replacements for the PROCESS-based mediation and moderation analyses. Based on the theoretical and empirical background reviewed above, this study proposed the hypothetical model shown in Figure 1 and tested the following hypotheses:

*H1*: Physical activity is positively associated with adolescents’ well-being.

*H2*: Cognitive reappraisal and psychological resilience mediate the association between physical activity and well-being.

*H3*: Loneliness moderates the association between psychological resilience and well-being.

## 2 Methods

### 2.1 Moderated mediation analysis

#### 2.1.1 Participants

This study used convenience sampling to recruit adolescents primarily from Sichuan and Yunnan Provinces in China. Eligible participants were aged 10–19 years, were able to read and complete a Chinese online questionnaire, and agreed to participate voluntarily. Data were collected through an anonymous questionnaire created on the Wenjuanxing platform. The survey link was disseminated through social media channels by the research team and local contacts rather than through a formal school-based sampling frame; therefore, schools did not provide sampling lists or select participants for inclusion. Before completing the questionnaire, participants were informed that the study was for research purposes only, that no personally identifiable information would be collected, and that they could withdraw before submission. For minor participants, adolescent assent and parental or guardian consent procedures were implemented in accordance with the approved ethics protocol. This study involving human participants was reviewed and approved by the Science and Technology Ethics Committee of Yunnan Minzu University (approval number: 2026015). Because the data were based on self-reports, procedural control measures were adopted during data collection following the recommendations of Memon et al. (2023). First, scales measuring different constructs were presented on separate questionnaire pages to reduce response consistency caused by the perceived connection among items. Second, a 15-s interval was set before participants could proceed across pages, which helped reduce overly rapid responding. A total of 1,156 questionnaires were collected. Responses were excluded if they met any of the preset invalidity criteria: total response time less than 1 min, obvious straight-line responding, such as selecting the same option for six or more consecutive scale items, anomalous key information, or signs of random or careless responding. After quality control, 964 valid responses were retained for analysis. Because invalid responses were removed according to these quality-control criteria and were not included in the analytic dataset, comparisons between retained and excluded cases were not conducted. The age range of the valid sample was 10–19 years, with a mean age of 15.23 years (SD = 3.32). The gender distribution was 596 males and 368 females, accounting for 61.83 and 38.17%, respectively.

#### 2.1.2 Research instruments

The level of physical activity participation was assessed using the Physical Activity Scale revised by Liang (1994). This scale measures adolescents’ organized physical activity participation over the past month in terms of frequency, intensity, and duration. The scale consists of three items rated on a 1–5-point scale. The total physical activity score is calculated as frequency × intensity × (duration – 1), with higher scores indicating greater participation in organized physical activity. According to the scoring criteria, scores of ≤19, 20–42, and ≥43 indicate low, moderate, and high levels of physical activity participation, respectively. Because the total score combines three behavioral dimensions—frequency, intensity, and duration—the scale was treated as a composite indicator of organized physical activity participation. In the present sample, Cronbach’s alpha was 0.602, the KMO statistic was 0.585, composite reliability was 0.628, and the average variance extracted was 0.381. Standardized factor loadings ranged from 0.35 to 0.79. Given that the scale contains only three items and the single-factor model was just-identified, global CFA fit indices were not used as primary evidence of model fit.

Cognitive reappraisal is measured via the cognitive reappraisal item of the Emotion Regulation Questionnaire for Children and adolescents (ERQ-CA) developed by Martín-Albo et al. (2020) which measures an individual’s capacity to regulate emotions by modifying one’s cognitive reappraisal of emotional occurrences. Participants scored each item on a 7-point Likert scale, ranging from 1 (strongly disagree) to 7 (strongly agree); Representative items include “when I want to feel less sad, I try to view things from another angle,” “I change my emotional state through the way I think,” etc., and higher scores mean that people use cognitive reappraisal (a positive emotion regulation strategy) more often and have stronger positive emotion regulation ability. The Cronbach’s alpha of the scale in this study was 0.945, and the KMO was 0.926. The results of the confirmatory factor analysis are as follows: χ^2^/df = 9.550, RMSEA = 0.094, SRMR = 0.016, GFI = 0.971, NFI = 0.984, CFI = 0.986, IFI = 0.986.

Psychological resilience was assessed using the unidimensional 10-item version of the Connor-Davidson Resilience Scale (CD-RISC) developed by Campbell-Sills and Stein (2007), which measures an individual’s capacity to cope with stress and adversity and to recover from difficult circumstances. Participants rated the items on a 0–4 scale (from strongly disagree to strongly agree), such as whether I can adapt to change, whether I can cope with all sorts of things coming their way, etc., and “handle bad moods.” A higher total score indicates greater psychological resilience. In this study, the Cronbach’s alpha coefficient for the scale was 0.968, and the KMO was 0.901. The results of the confirmatory factor analysis are as follows: χ^2^/df = 16.619, RMSEA = 0.127, SRMR = 0.029, GFI = 0.949, NFI = 0.946, CFI = 0.949, IFI = 0.949.

Loneliness was assessed using the Revised UCLA Loneliness Scale – 6-item version (Wongpakaran et al., 2020), which measures individuals’ subjective experiences of loneliness, exclusion, and lack of companionship in social interactions. Participants rated items on a 4-point Likert scale ranging from 1 (Never) to 4 (Often), with representative questions such as “How often do you feel lonely?” and “How often do you feel that no one truly understands you?” A higher total score indicates a stronger experience of loneliness. In this study, the scale’s Cronbach’s alpha coefficient was 0.923, and the KMO was 0.900. The results of the confirmatory factor analysis are as follows: χ^2^/df = 26.901, RMSEA = 0.163, SRMR = 0.034, GFI = 0.920, NFI = 0.946, CFI = 0.948, IFI = 0.948.

Well-being was assessed using the Short Warwick–Edinburgh Mental Well-Being Scale (SWEMWBS) developed by Stewart-Brown et al. (2009). The scale evaluates positive aspects of well-being, including optimism, self-worth, emotional relaxation, and problem-solving ability. Participants rated items on a 5-point Likert scale ranging from 1 (Never) to 5 (Always), such as “I feel optimistic about the future” and “I feel useful.” A higher total score indicates a higher level of well-being. In this study, the scale’s Cronbach’s alpha coefficient was 0.928, and the KMO was 0.929. The results of the confirmatory factor analysis are as follows: X^2^/df = 11.686, RMSEA = 0.105, SRMR = 0.029, GFI = 0.948, NFI = 0.966, CFI = 0.969, IFI = 0.969.

Across the psychological scales, internal consistency and convergent validity were generally acceptable, as indicated by Cronbach’s alpha coefficients, KMO values, composite reliability, average variance extracted, and standardized factor loadings. However, the CFA results showed mixed model fit for several scales. Specifically, although CFI, IFI, and SRMR suggested acceptable or adequate fit, the RMSEA values were relatively high for psychological resilience, loneliness, and well-being. These results indicate that the one-factor structures captured the main common variance of the scales but may not fully account for local item-level dependencies. Therefore, the measurement results were interpreted based on multiple fit indices rather than relying on a single fit criterion. The original one-factor structures of the established scales were retained to preserve scale comparability, and no *post-hoc* item deletion or correlated residuals were introduced solely to improve model fit.

### 2.2 Machine learning methods

To complement the PROCESS-based mediation and moderation analyses, we used XGBoost regression to characterize the predictive structure of adolescent well-being in a flexible nonlinear framework. This analysis extended the regression-based models by identifying the relative contribution of each predictor and by describing how predicted well-being varied across different levels and combinations of key variables. The outcome variable was the same total well-being score used in the mediation models. Predictor variables included demographic and activity-related variables (age, gender, exercise experience, and physical activity score) as well as theoretically proximal psychosocial variables (psychological resilience, cognitive reappraisal, and loneliness), allowing the model to compare the relative predictive contribution of lifestyle and psychological factors within the same analytic framework. Before model fitting, all selected variables were converted into numeric format, and cases with missing values were removed. The final analytic sample was randomly divided into a training set and a test set using an 80:20 split. A fixed random seed of 20,260,319 was used to ensure reproducibility. Model development was conducted in the training set, whereas final predictive performance was evaluated in the independent test set.

Hyperparameter tuning was conducted using five-fold cross-validation within the training set. The tuning grid included maximum tree depth, learning rate, minimum child weight, subsampling ratio, column sampling ratio, gamma, and the number of boosting rounds. Specifically, max_depth was tuned across 2, 3, and 4; eta across 0.05 and 0.10; min_child_weight across 1 and 3; subsample across 0.80 and 1.00; colsample_bytree across 0.80 and 1.00; gamma across 0 and 0.10; and nrounds across 100 and 200. Early stopping was applied after 20 rounds without improvement. The final XGBoost model was selected according to the lowest cross-validated RMSE and then refitted on the full training set. Model performance was evaluated using RMSE, MAE, and R^2^ in the training and test sets. Model interpretation was conducted using SHAP values derived from the native XGBoost prediction-contribution function. Mean absolute SHAP values were used to summarize each predictor’s average contribution to model prediction. SHAP dependence plots and partial dependence plots were then used to describe nonlinear predictive patterns and conditional differences across levels of physical activity, cognitive reappraisal, psychological resilience, and loneliness.

### 2.3 Data analysis

Data analyses were conducted using SPSS 27.0, PROCESS 4.2, AMOS 24.0, and R 4.3.2. SPSS was used for descriptive statistics, correlation analysis, reliability testing, regression diagnostics, and sensitivity analyses. AMOS was used for confirmatory factor analysis. PROCESS Model 6 was used to test the sequential mediation model, and PROCESS Model 87 was used to test the moderated mediation model. Continuous study variables were standardized before the mediation and moderated mediation analyses, and interaction terms were computed from standardized variables. Indirect effects were evaluated using 5,000 bootstrap samples and 95% percentile bootstrap confidence intervals. Age, gender, and sport experience were included as covariates because adolescent well-being, physical activity participation, and psychological resources may vary by developmental stage, sex, and prior sport exposure. Regression assumptions were evaluated by examining multicollinearity, residual patterns, influential observations, residual distribution, and heteroscedasticity. Variance inflation factors were used to assess multicollinearity, Cook’s distance and studentized residuals were used to identify influential observations, and heteroscedasticity-consistent standard errors were used in sensitivity checks. Supplementary machine learning analyses were conducted in R using xgboost, readxl, openxlsx, dplyr, and ggplot2 for model training, data processing, result export, and visualization.

## 3 Results

### 3.1 Common method bias

Harman’s single-factor test was used to assess common method bias (Podsakoff and Organ, 1986). The results indicated that among the five factors with eigenvalues greater than one obtained without rotation, the first factor explained 38.87% of the variance, which was below the commonly used 40% threshold. Thus, Harman’s single-factor test did not indicate the presence of a dominant common factor; however, residual common method variance cannot be ruled out.

### 3.2 Descriptive statistics and correlation analysis

As shown in Table 1, physical activity was significantly and positively correlated with cognitive reappraisal, psychological resilience, and well-being (*r* = 0.117, 0.115, and 0.126, respectively; all *p* < 0.01). These correlations were statistically reliable but small in magnitude. In contrast, well-being showed stronger associations with cognitive reappraisal (*r* = 0.513), psychological resilience (*r* = 0.519), and loneliness (*r* = −0.462). Loneliness was also negatively correlated with cognitive reappraisal and psychological resilience (*r* = −0.359 and −0.222, respectively; both *p* < 0.01). These results indicate that physical activity was modestly associated with psychological resources and well-being, whereas proximal psychological variables showed stronger associations with well-being.

**Table 1:** Descriptive statistics and correlation analysis (*n* = 964).

Variable	1	2	3	4	5	6	7	8
1. PA	1							
2. CR	0.117^**^	1						
3. PR	0.115^**^	0.347^**^	1					
4. LN	−0.080^*^	−0.359^**^	−0.222^**^	1				
5. WB	0.126^**^	0.513^**^	0.519^**^	−0.462^**^	1			
6. Age	0.000	−0.016	0.035	0.091^**^	−0.013	1		
7. Gender	−0.234^**^	−0.111^**^	−0.025	0.151^**^	−0.125^**^	−0.107^**^	1	
8. SE	0.091^**^	0.064^*^	0.017	0.010	0.094^**^	0.163^**^	−0.186^**^	1
M	21.12	23.98	23.29	9.99	23.85	15.23	1.38	9.89
SD	17.71	23.29	11.09	4.269	4.759	3.32	0.48	11.95

^*^ denotes *p* < 0.05, ^**^ denotes *p* < 0.01, ^***^ denotes *p* < 0.001. PA, physical activity; CR, cognitive reappraisal; PR, psychological resilience; LN, loneliness; WB, well-being; SE, sports experience; M, mean; SD, standard deviation (same below).

### 3.3 Testing for mediating effects

**Figure 2:**
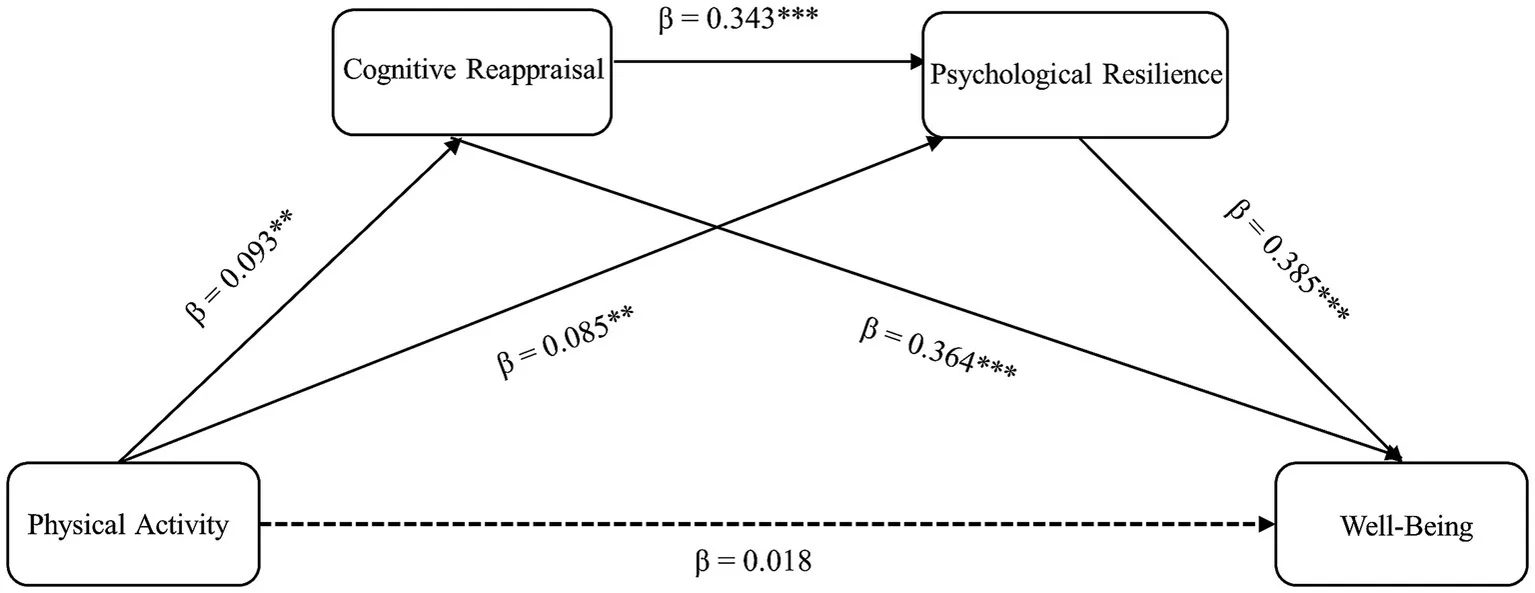
The chained mediating effects of cognitive reappraisal and psychological resilience on the association between physical activity and adolescent well-being. **denotes *p* < 0.01, ***denotes *p* < 0.001.

All variables were standardized before analysis, and age, gender, and sport experience were included as covariates. Using PROCESS Model 6, we tested cognitive reappraisal and psychological resilience as sequential mediators of the association between physical activity and well-being. As shown in Figure 2 and Table 2, the standardized total effect of physical activity on well-being was significant (*β* = 0.098, 95% CI [0.032, 0.169]), whereas the standardized direct effect was not significant after cognitive reappraisal and psychological resilience were included (*β* = 0.018, 95% CI [−0.034, 0.074]). The standardized total indirect effect was significant (*β* = 0.079, 95% CI [0.037, 0.124]). Significant indirect associations were observed through cognitive reappraisal (*β* = 0.034, 95% CI [0.010, 0.061]), through psychological resilience (*β* = 0.033, 95% CI [0.006, 0.061]), and through the sequential pathway from cognitive reappraisal to psychological resilience (*β* = 0.012, 95% CI [0.004, 0.024]). Although these indirect effects were statistically significant, their magnitudes were modest. These results indicate that physical activity was positively associated with well-being at the total-effect level, but that this association was mainly reflected through indirect pathways involving cognitive reappraisal and psychological resilience rather than through a significant direct pathway.

**Table 2:** Chain mediation effects.

Effect	Effect size (β)	BootSE	BootLLCI	BootULCI
Total Effect	0.098	0.035	0.032	0.169
Direct Effect	0.018	0.028	−0.034	0.074
Total Indirect Effect	0.079	0.022	0.037	0.124
Indirect Effect 1: (PA → CR → WB)	0.034	0.013	0.010	0.061
Indirect Effect 2: (PA → PR → WB)	0.033	0.014	0.006	0.061
Indirect Effect 3: (PA → CR → PR → WB)	0.012	0.005	0.004	0.024

### 3.4 Moderated chain mediation analysis

**Figure 3:**
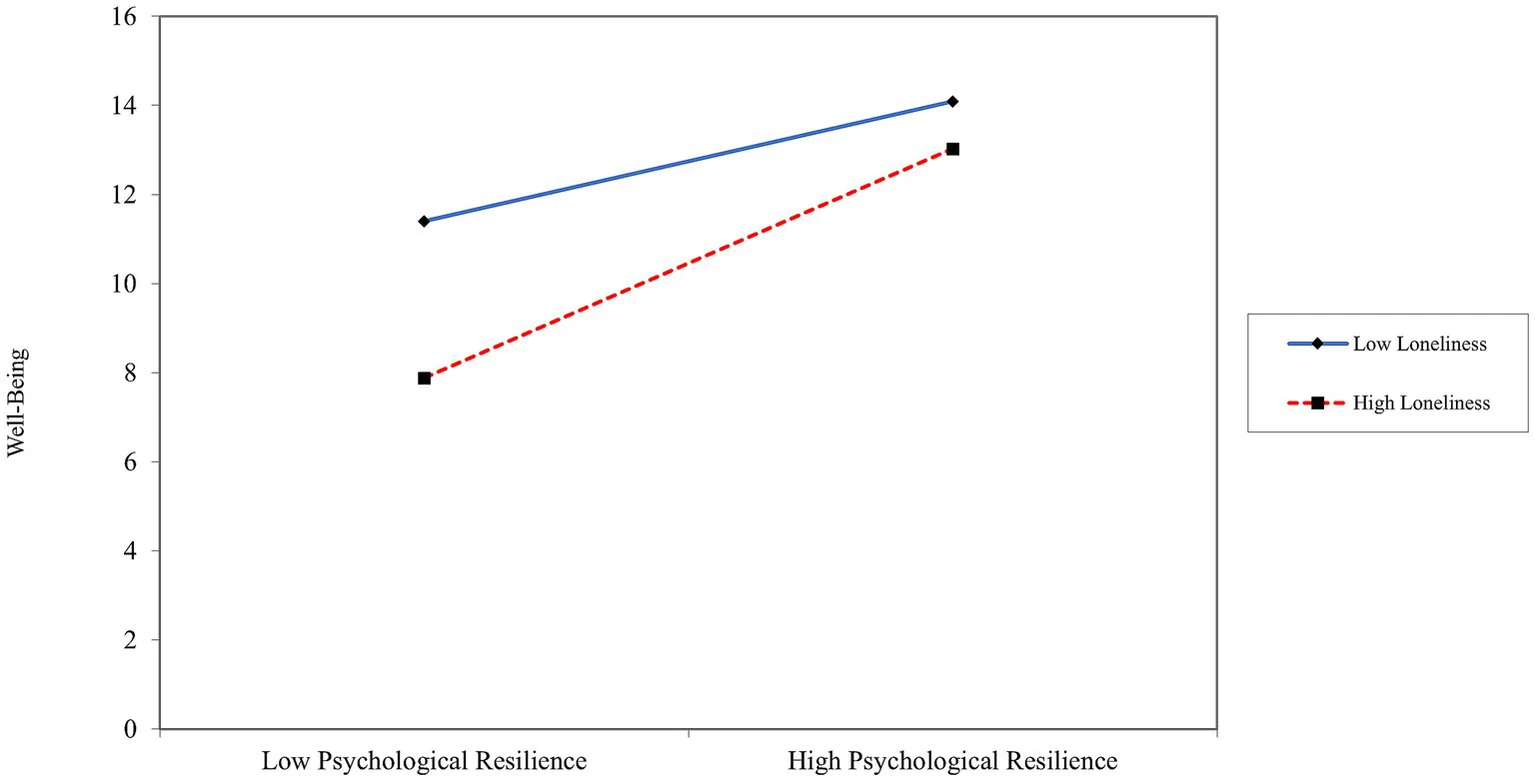
The moderating role of loneliness in the association between psychological resilience and adolescent well-being.

PROCESS Model 87 was used to examine whether loneliness moderated the resilience-well-being association within the chained mediation model. The variance inflation factors (VIF) for all predictor variables in this study ranged from 1.003 to 1.263, all of which were below the critical value of 5, indicating that there were no serious multicollinearity issues (Hair et al., 2010). The results show (see Table 3) that psychological resilience positively predicts well-being (*β* = 0.41, *t* = 14.88, *p* < 0.001), and the interaction term between psychological resilience and loneliness is significant (*β* = 0.13, *t* = 4.97, *p* < 0.001), indicating that loneliness significantly moderates the effect of psychological resilience on well-being. To gain a clearer understanding of the moderating effect of loneliness, the standardized loneliness scores were divided into high and low groups based on ±1 standard deviation, and a simple slope test was conducted; the results are shown in Figure 3. Compared with the low loneliness group (*β* = 0.29, *t* = 10.28, *p* < 0.001), the predictive effect of psychological resilience on well-being was stronger in the high loneliness group (*β* = 0.54, *t* = 11.96, *p* < 0.001). This pattern indicates that the resilience-well-being association was stronger among adolescents reporting higher loneliness. Therefore, H3 was supported.

**Table 3:** Moderated chain mediation effects.

Predictor variables	Equation 1: CR		Equation 2: PR			Equation 3: WB				Equation 4: WB		
	*β*	*SE*	*t*	*β*	*SE*	*t*	*β*	*SE*	*t*	*β*	*SE*	*t*
PA	0.12	0.03	3.66***	0.08	0.03	2.49*	0.04	0.03	1.49	0.03	0.02	1.27
CR				0.34	0.03	11.15***	0.38	0.03	13.98***	0.27	0.03	9.95***
PR							0.39	0.03	14.34***	0.41	0.03	14.88***
LN										−0.24	0.03	−9.15***
LN										0.13	0.03	4.97***
R^2^	0.01		0.13			0.4				0.48		
F	13.38***		69.46***			210.43***				174.36***		

Equation 3 represents the model without moderator variables and interaction terms, while Equation 4 represents the full model that includes moderator variables and interaction terms. *denotes *p* < 0.05, *** denotes *p* < 0.001.

We further examined the moderating effect of loneliness on the mediating pathway. The indirect association between physical activity and well-being via psychological resilience increased across loneliness levels: low loneliness (Effect = 0.022, 95% CI [0.003, 0.044]), moderate loneliness (Effect = 0.031, 95% CI [0.004, 0.060]), and high loneliness (Effect = 0.041, 95% CI [0.005, 0.080]). The moderated mediation index was significant (Index = 0.010, 95% CI [0.001, 0.022]), indicating that loneliness positively moderated the mediating effect of psychological resilience in the relationship between physical activity and well-being. The indirect effect of the chained mediation pathway (physical activity → cognitive reappraisal → psychological resilience → well-being) also increased at higher levels of loneliness: low loneliness (Effect = 0.012, 95% CI [0.005, 0.021]), moderate loneliness (Effect = 0.016, 95% CI [0.007, 0.028]) and high loneliness (Effect = 0.021, 95% CI [0.009, 0.038]). The moderated mediation index for the chained pathway was also significant (Index = 0.005, 95% CI [0.002, 0.010]), indicating that loneliness conditioned the sequential indirect association linking physical activity, cognitive reappraisal, psychological resilience, and well-being.

Sensitivity analyses were conducted to examine the robustness of the regression-based findings. The moderated regression model was first estimated with and without covariates. The core pattern remained consistent: cognitive reappraisal, psychological resilience, loneliness, and the interaction between psychological resilience and loneliness remained significant predictors of well-being, whereas the direct association between physical activity and well-being remained small and non-significant after these psychological variables were included. When age, gender, and sport experience were included as covariates, the model explained 48.2% of the variance in well-being. Treating physical activity as a categorical variable based on low, moderate, and high activity levels produced the same substantive pattern. Additional checks using heteroscedasticity-consistent standard errors and models excluding influential observations also supported the stability of the main conclusions. These analyses indicated that the primary findings were not dependent on a single covariate specification or on treating physical activity only as a continuous score.

### 3.5 Machine learning results

The XGBoost model provided acceptable internal predictive performance for adolescent well-being. During five-fold cross-validation in the training set, the best parameter combination yielded a cross-validated RMSE of 3.448 (SD = 0.290). After refitting the final model on the full training set, the model yielded an RMSE of 3.067, an MAE of 2.170, and an R^2^ of 0.600 in the training set. In the independent test set, the model yielded an RMSE of 2.599, an MAE of 1.860, and an R^2^ of 0.640 (see Table 4). The slightly higher test-set R^2^ may reflect random variation in the 80:20 partition and the possibility that the test set contained observations that were comparatively easier to predict.

**Table 4:** Predictive performance of the XGBoost model on the training set and the independent test set.

Dataset	Sample size	RMSE	MAE	R^2^
Training set	771.000	3.067	2.170	0.600
Test set	193.000	2.599	1.860	0.640

**Figure 4:**
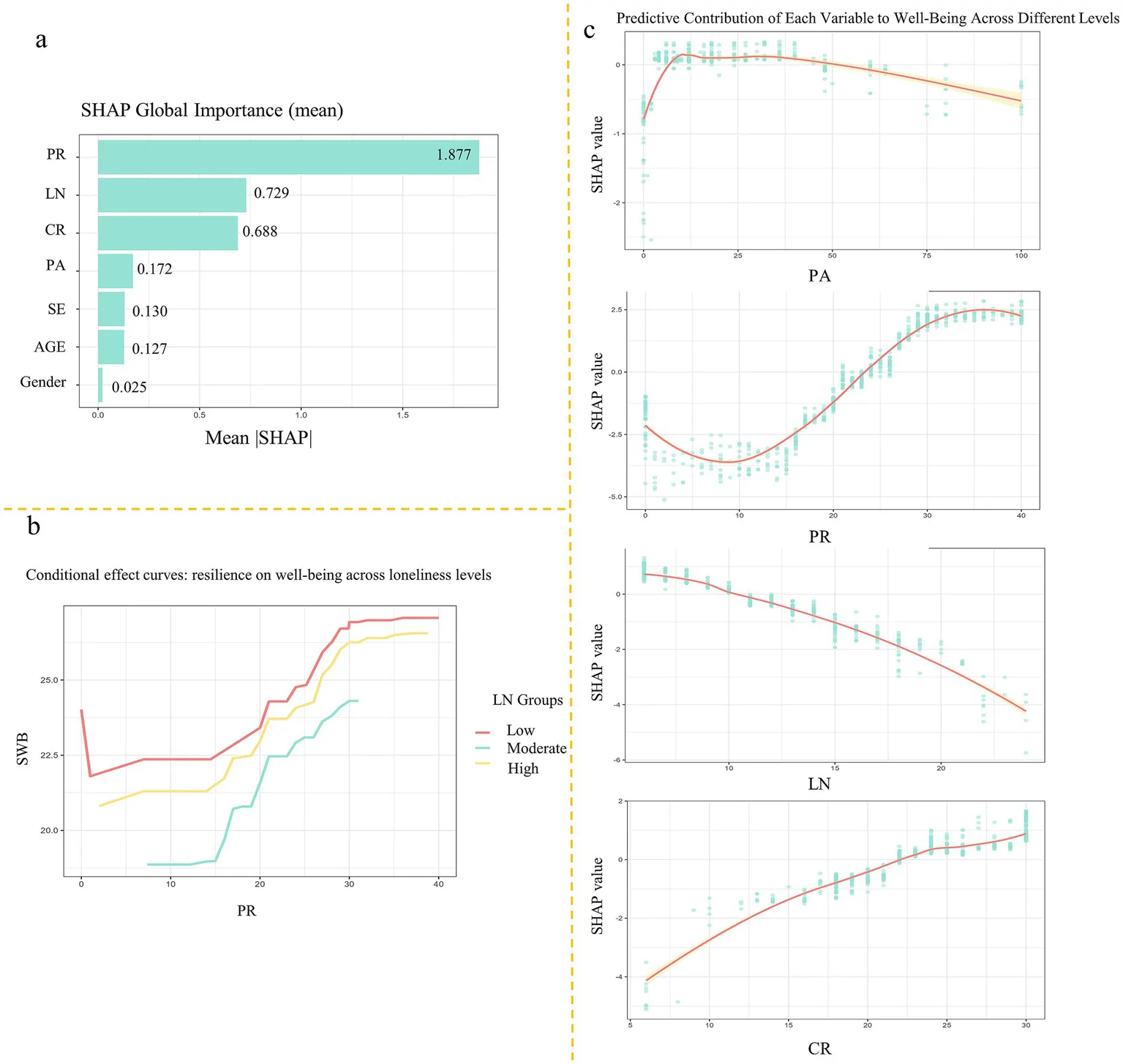
Global SHAP importance, dependence patterns, and conditional association curves for well-being prediction. **(a)** Mean absolute SHAP values showing the global predictive importance of all variables. **(b)** Conditional effect curves showing the association between psychological resilience and predicted well-being across low, moderate, and high levels of loneliness. **(c)** SHAP dependence plots showing the predictive contributions of physical activity, psychological resilience, loneliness, and cognitive reappraisal to well-being.

The SHAP analysis showed a clear hierarchy of predictive contributions in the XGBoost model. Psychological resilience had the largest mean absolute SHAP value (1.877), followed by loneliness (0.729), cognitive reappraisal (0.688), physical activity (0.172), exercise experience (0.130), age (0.127), and gender (0.025). This pattern indicates that proximal psychological variables contributed more strongly to well-being prediction than physical activity alone. The partial dependence results further showed nonlinear predictive patterns. Predicted well-being decreased as loneliness increased, increased with cognitive reappraisal, and increased more strongly with psychological resilience. Physical activity showed a smaller nonlinear pattern, with predicted well-being increasing from low activity levels and then tending to plateau (see Figure 4).

The stratified partial dependence results further clarified how loneliness shaped the prediction of well-being. Across loneliness groups, higher psychological resilience corresponded to higher predicted well-being. However, adolescents with higher loneliness showed lower overall predicted well-being, and the increase associated with resilience was especially visible in this group. This pattern complements the moderated mediation results by showing how the resilience-well-being association appeared across different loneliness levels in the fitted XGBoost model.

**Figure 5:**
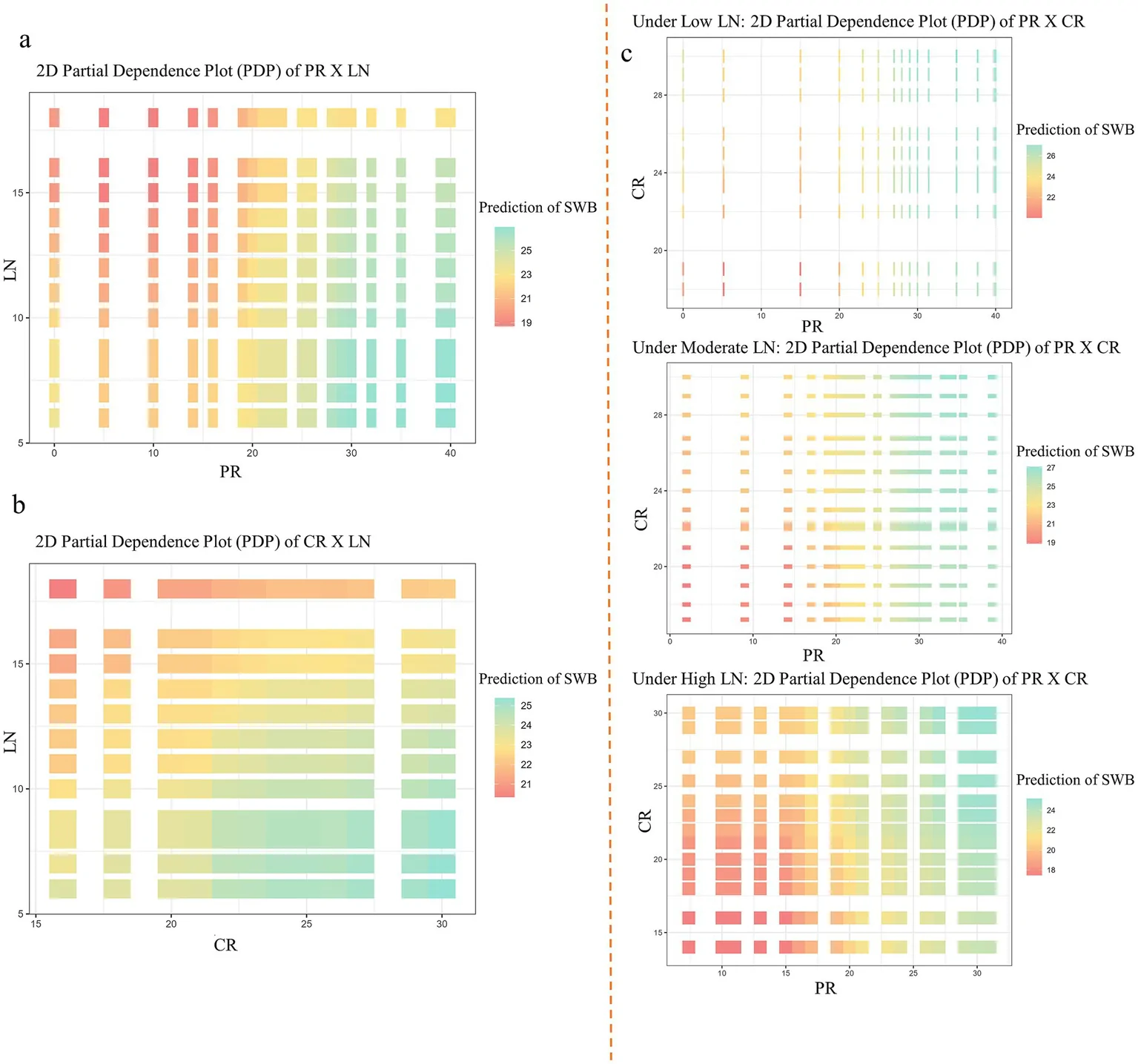
Two-dimensional partial dependence plots of key predictor combinations for well-being. **(a)** Joint partial dependence of psychological resilience and loneliness. **(b)** Joint partial dependence of cognitive reappraisal and loneliness. **(c)** Joint partial dependence of psychological resilience and cognitive reappraisal under low, moderate, and high levels of loneliness.

The results of the two-dimensional partial dependence analysis further reinforce this finding. Figure 5a shows that the combination of psychological resilience and loneliness forms a clear gradient distribution, in which high psychological resilience and low loneliness correspond to the highest predicted levels of well-being, while low psychological resilience and high loneliness correspond to the lowest predicted levels of well-being. This indicates that well-being is not determined independently by a single variable, but rather exhibits distinct conditional differences under the combined influence of psychological resources and risk states. Figure 5b further indicates that the combination of cognitive reappraisal and loneliness also exhibits a similar trend, namely, higher levels of cognitive reappraisal and lower levels of loneliness jointly correspond to higher predicted levels of well-being. Figure 5c suggests that under different conditions of loneliness, the joint predictive pattern of psychological resilience and cognitive reappraisal shows further differentiation, with the increase in predicted well-being being more pronounced when both factors are elevated under conditions of high loneliness. Although these higher-order combination results are primarily exploratory in nature, they still suggest that the predictive structure of well-being possesses a complexity that goes beyond simple linear superposition.

Taken together, the machine learning results extend the PROCESS-based findings by showing how well-being prediction varies across nonlinear and conditional patterns. Psychological resilience made the largest model-based predictive contribution in the XGBoost model, while loneliness and cognitive reappraisal also contributed substantially. Physical activity made a smaller contribution, and its partial dependence curve showed a modest increase in predicted well-being from low to moderate activity levels followed by a plateau. These results refine the interpretation of the mediation findings by showing that psychological resources occupy a more proximal position in the prediction of adolescent well-being.

## 4 Discussion

### 4.1 Overview of findings

This study examined how physical activity was associated with adolescent well-being through cognitive reappraisal and psychological resilience, and whether loneliness moderated these associations. Physical activity was not directly associated with well-being after the mediators were included. Instead, the association was reflected mainly through independent and sequential indirect pathways involving cognitive reappraisal and psychological resilience. Loneliness moderated the association between psychological resilience and well-being and further conditioned the indirect pathways involving resilience. The machine learning analysis provided a complementary predictive description of nonlinear and conditional patterns in well-being. Psychological resilience made the largest model-based predictive contribution in the XGBoost model, followed by loneliness and cognitive reappraisal, whereas physical activity showed a smaller nonlinear contribution. Overall, the findings suggest that adolescent well-being is more closely linked to proximal psychological resources than to physical activity alone, especially under conditions of higher loneliness.

### 4.2 Chain mediation analysis

The present study found that physical activity was related to adolescent well-being mainly through three statistically supported indirect associations: the association through cognitive reappraisal, the association through psychological resilience, and the sequential association involving cognitive reappraisal and psychological resilience. Among these pathways, the indirect association through cognitive reappraisal was larger than that through psychological resilience, suggesting that emotion regulation may represent a particularly salient psychological correlate in the link between physical activity and well-being.

This finding is consistent with emotion regulation theory. Gross (2015). argued that cognitive reappraisal is a central process through which individuals regulate emotional responses to challenging situations. Physical activity contexts may involve fatigue, frustration, competition, and performance-related difficulties, which may make adaptive cognitive reinterpretation especially relevant (Jones and Sheffield, 2007). Adolescents who report more frequent use of cognitive reappraisal may also report more flexible interpretations of stressors, together with higher levels of resilience and well-being. From this perspective, cognitive reappraisal and psychological resilience can be understood as closely related psychological resources in the physical activity–well-being association.

The non-significant direct association between physical activity and well-being after including the mediators further suggests that the association in this sample was mainly reflected through indirect pathways. This pattern aligns with the resource-gain perspective, which suggests that behavioral and psychological resources may accumulate or cluster together in adaptive functioning (Hobfoll, 2002). Physical activity may therefore be more closely linked to adolescent well-being when considered alongside psychological resources such as adaptive emotion regulation and resilience. This interpretation highlights the importance of considering the psychological context of physical activity rather than focusing only on participation level. Recent evidence also suggests that the association between physical activity and mental health may involve both biological and psychological processes, including neurobiological adaptation, stress regulation, resilience, and adaptive coping (Kanani et al., 2025). In the present study, cognitive reappraisal and psychological resilience may therefore be understood as proximal psychological resources that help explain why physical activity was indirectly associated with adolescent well-being.

### 4.3 Analysis of moderated chain mediation

The present study found that loneliness significantly moderated the association between psychological resilience and well-being. Specifically, the positive association between resilience and well-being was stronger among adolescents with higher levels of loneliness than among those with lower loneliness. This suggests that loneliness may function as an important boundary condition, with the resilience-well-being association becoming more pronounced when adolescents experience greater social disconnection. This pattern can be understood from a compensatory resource perspective. Adolescents with high loneliness may have less access to external interpersonal resources, making internal psychological resources such as resilience relatively more important for maintaining well-being. In contrast, for adolescents with lower loneliness, social connectedness may provide additional support, thereby reducing the relative contribution of resilience. This interpretation is broadly consistent with prior research suggesting that internal psychological resources may show stronger protective associations under conditions of greater loneliness or reduced social support (Cohen and Wills, 1985; Skoko et al., 2024).

More importantly, the moderating role of loneliness extended beyond the direct resilience to well-being association to the indirect pathways linking physical activity and well-being. The indirect effect through psychological resilience became stronger as loneliness increased, and the same pattern was observed for the chained mediation pathway through cognitive reappraisal and psychological resilience. These findings suggest that loneliness not only conditioned the resilience-well-being association, but also shaped the strength of the indirect associations linking physical activity with well-being through psychological resources. From the perspective of resource gain, this pattern may indicate that psychological resources linked to physical activity are more strongly associated with well-being when external social resources are limited (Hobfoll, 2002). The supplementary machine learning analysis similarly described a steeper resilience-well-being predictive pattern at higher levels of loneliness, providing a more fine-grained predictive description of this boundary condition. Together, these findings point to loneliness as a meaningful context in which future research can examine how activity-related psychological resources are connected with adolescent well-being.

### 4.4 Machine learning

The machine learning analysis provided a more detailed predictive description of how key variables were related to adolescent well-being. This use of explainable machine learning is consistent with recent work in physical activity and adolescent health research, where machine learning has been used to classify movement behaviors, process accelerometer-derived activity information, and identify heterogeneous predictive patterns that are not easily summarized by conventional regression models (Kadirvelu et al., 2025; Sameh et al., 2025; Thornton et al., 2022). In the present study, XGBoost captured nonlinear and conditional predictive patterns that were not directly summarized by the PROCESS model. Psychological resilience had the largest model-based predictive contribution, followed by loneliness and cognitive reappraisal, whereas physical activity contributed less directly to prediction. This pattern paralleled the mediation findings in emphasizing proximal psychological resources, while adding a predictive description of how well-being varied across different levels and combinations of these variables.

The partial dependence results also suggested that the model-based association between physical activity and predicted well-being was not simply linear. Predicted well-being increased from low activity levels and then tended to plateau, but the overall contribution of physical activity was smaller than that of psychological resilience, loneliness, and cognitive reappraisal. This pattern describes a fitted predictive profile rather than a longitudinal dose–response pattern. It suggests that physical activity may be more closely related to well-being when considered alongside psychological resources, rather than as a strong independent predictor.

The machine learning analysis also showed that the resilience to well-being prediction varied across loneliness levels. Adolescents with higher loneliness had lower predicted well-being overall, but predicted well-being still increased as resilience increased. This pattern paralleled the moderated mediation result and suggests that resilience was especially informative for predicting well-being among adolescents reporting higher loneliness. However, the observed effect sizes do not by themselves establish whether resilience-focused or activity-based programs would produce meaningful improvements in practice. Future intervention studies are needed to test whether strengthening psychological resources in physical activity contexts can generate practically meaningful changes in adolescent well-being.

### 4.5 Theoretical contributions and practical implications

This study makes two main theoretical contributions. First, it shows that physical activity was statistically linked to adolescent well-being mainly through cognitive reappraisal and psychological resilience rather than a direct pathway, which helps clarify the psychological correlates linking activity to well-being. Second, it identifies loneliness as an important boundary condition and further uses machine learning to describe nonlinear predictive patterns among these variables. In particular, psychological resilience showed the largest model-based predictive contribution to well-being, which refines the description of how different factors were related in the present sample.

The findings offer a measured basis for future applied research. Physical activity showed small but significant associations with cognitive reappraisal, psychological resilience, and well-being, whereas psychological resources showed stronger associations with well-being. This pattern suggests that the value of physical activity for adolescent well-being may depend partly on the psychological resources that accompany activity experiences. Future school- or community-based studies could examine whether physical education classes, extracurricular sports, or group-based activity programs that include emotion-regulation practice, resilience-building activities, and supportive peer interaction are more closely linked to adolescent well-being than activity participation alone. For adolescents reporting higher loneliness, future research may also test whether activity contexts that provide structured social connection and psychological resource training are especially relevant. Such work would help clarify whether and how physical activity can be embedded within broader approaches to adolescent mental health promotion.

### 4.6 Limitations and future directions

This study has several limitations. First, the cross-sectional design limits conclusions about temporal ordering and causality among physical activity, cognitive reappraisal, psychological resilience, loneliness, and well-being. Although the proposed model was theoretically grounded, the observed indirect pathways should be understood as statistical associations within the current sample. Future longitudinal, experimental, and ecological momentary assessment studies are needed to clarify the temporal dynamics among these variables. Second, all variables were measured using adolescent self-report questionnaires, which may increase the risk of common method influences and social desirability bias. Although procedural controls and Harman’s single-factor diagnostic were used, residual common method variance cannot be ruled out. Future studies should combine self-report measures with objective physical activity indicators and parent-, teacher-, or multi-wave data. Third, the physical activity measure was a brief three-item behavioral composite with modest internal consistency and convergent validity. Findings involving physical activity should therefore be interpreted as associations based on a concise self-report index rather than a comprehensive assessment of physical activity. Fourth, the sample was recruited online using convenience sampling and was primarily drawn from Sichuan and Yunnan Provinces in China, which may limit generalizability. The study also did not collect detailed information on socioeconomic background, sleep quality, depressive symptoms, anxiety symptoms, academic stress, family support, body composition, or other health-related characteristics. These unmeasured factors could not be used to evaluate subgroup differences or adjust for additional potential confounding influences. Fifth, although the machine learning analysis described nonlinear and heterogeneous predictive patterns, the model was evaluated only through internal validation within the current sample. The XGBoost and SHAP results should not be interpreted as causal evidence or as evidence of external generalizability. Future studies should test the model in independent adolescent samples from different regions and school contexts.

## 5 Conclusion

In this cross-sectional sample of Chinese adolescents, physical activity was indirectly associated with well-being through cognitive reappraisal and psychological resilience, whereas its direct association with well-being was not significant after these psychological resources were included. Loneliness conditioned the association between psychological resilience and well-being, with a stronger resilience-well-being association observed among adolescents reporting higher loneliness. Supplementary machine learning analyses further showed that psychological resilience made the largest model-based predictive contribution to well-being, followed by loneliness and cognitive reappraisal, while physical activity showed a smaller and nonlinear predictive contribution. Together, these findings suggest that adolescent well-being is more closely related to proximal psychological resources than to physical activity alone, and that loneliness may shape the strength of these associations. Because the evidence is based on a cross-sectional observational design, the findings should be interpreted as statistical and predictive associations rather than causal effects. Longitudinal and intervention studies are needed to clarify the temporal ordering of these variables and to test whether physical activity contexts that support cognitive reappraisal and psychological resilience can contribute to meaningful improvements in adolescent well-being.

## Data Availability

The datasets generated and analyzed during the current study are not publicly available because they contain participant-level questionnaire data from minors and may raise privacy and ethical concerns. De-identified data supporting the findings of this study may be made available from the authors upon reasonable request, subject to institutional approval and compliance with relevant ethical requirements. Requests to access the datasets should be directed to Longjiang Chen at longjiang-chen@outlook.com.
